# Micro RNA-124a Regulates Lipolysis via Adipose Triglyceride Lipase and Comparative Gene Identification 58

**DOI:** 10.3390/ijms16048555

**Published:** 2015-04-16

**Authors:** Suman K. Das, Elke Stadelmeyer, Silvia Schauer, Anna Schwarz, Heimo Strohmaier, Thiery Claudel, Rudolf Zechner, Gerald Hoefler, Paul W. Vesely

**Affiliations:** 1 Institute of Pathology, Medical University of Graz, Auenbruggerplatz 25, 8036 Graz, Austria; E-Mails: paramount.suman@gmail.com (S.K.D.); elke.stadelmeyer@medunigraz.at (E.S.); silvia.schauer@medunigraz.at (S.S.); anna.schwarz@medunigraz.at (A.S.); 2 Center for Medical Research, Medical University of Graz, Stiftingtalstrasse 24, 8010 Graz, Austria; E-Mail: heimo.strohmaier@medunigraz.at; 3 Hans Popper Laboratory of Molecular Hepatology, Division of Gastroenterology and Hepatology, Department of Internal Medicine III, Medical University of Vienna, Währinger Gürtel 18-20, 1090 Vienna, Austria; E-Mail: thierry.claudel@meduniwien.ac.at; 4 Institute of Molecular Biosciences, Karl Franzens University of Graz, Heinrichstraße 31, 8010 Graz, Austria; E-Mail: rudolf.zechner@uni-graz.at

**Keywords:** miR-124a, metabolism, lipolysis, ATGL, CGI-58

## Abstract

Lipolysis is the biochemical pathway responsible for the catabolism of cellular triacylglycerol (TG). Lipolytic TG breakdown is a central metabolic process leading to the generation of free fatty acids (FA) and glycerol, thereby regulating lipid, as well as energy homeostasis. The precise tuning of lipolysis is imperative to prevent lipotoxicity, obesity, diabetes and other related metabolic disorders. Here, we present our finding that miR-124a attenuates RNA and protein expression of the major TG hydrolase, adipose triglyceride lipase (ATGL/PNPLA2) and its co-activator comparative gene identification 58 (CGI-58/ABHD5). Ectopic expression of miR-124a in adipocytes leads to reduced lipolysis and increased cellular TG accumulation. This phenotype, however, can be rescued by overexpression of truncated *Atgl* lacking its 3'UTR, which harbors the identified miR-124a target site. In addition, we observe a strong negative correlation between miR-124a and *Atgl* expression in various murine tissues. Moreover, miR-124a regulates the expression of *Atgl* and *Cgi-58* in murine white adipose tissue during fasting as well as the expression of *Atgl* in murine liver, during fasting and re-feeding. Together, these results point to an instrumental role of miR-124a in the regulation of TG catabolism. Therefore, we suggest that miR-124a may be involved in the regulation of several cellular and organismal metabolic parameters, including lipid storage and plasma FA concentration.

## 1. Introduction

### 1.1. Lipases and Lipolysis

Fatty acids (FA) are essential energy substrates, precursors for membrane lipids, and signaling molecules. In cells, FA are stored in triacylglycerol (TG) within cytoplasmic lipid droplets (LD). Release of FA from LD requires the hydrolysis of TG by specific TG hydrolases (lipases). The complete, sequential hydrolysis of TG is dependent on three enzymes namely, adipose triglyceride lipase (ATGL), hormone-sensitive lipase (HSL), and monoglyceride lipase (MGL). The first step, which is considered rate limiting, is carried out by ATGL that converts TG to diacylglycerol (DG). HSL is mainly responsible for the hydrolysis of DG, and MGL eventually hydrolyzes monoacylglyceride (MG), yielding glycerol and FA [1,2]. To achieve its full hydrolytic activity ATGL requires comparative gene identification 58 (CGI-58, also known as ABHD5) as co-activator [3,4]. In humans [5] and mice [6], absence of ATGL drastically reduces TG hydrolysis. Several mutations of *CGI-58* lead to massive neutral lipid storage in tissues and additionally defective epidermal function of the skin. Accordingly, the resulting disease is called neutral lipid storage disease with ichthyosis (NLSDI) [7,8]. In humans, HSL null mutation is associated with insulin resistance [9]. Whereas, lack of ATGL activity has severe effects, lack of HSL or MGL causes relatively benign phenotypes, underlining the important biological role of ATGL [10,11,12,13].

### 1.2. Regulation of Lipases by miRNAs

Although, mechanisms for pre- and post- translational control of lipases are mostly unknown, they are conceivably of particular importance in various physiological situations like fasting, feeding or exercise [1] as well as in pathological conditions like cancer [14]. Parra *et al.*, presented evidence that miR-222 might target ATGL along with Peroxisome proliferator-activated receptor γ2 (PPARγ2), Fatty acid synthase (FASN), Stearoyl-CoA desaturase-1 (SCD1), HSL, and a few other genes involved in both adipogenesis and lipolysis [15]. Their conclusions were based on correlational evidence and did not include direct experimental proof for regulation of ATGL by miR-222.

We wondered if miRNAs could directly influence lipolysis by regulating the expression of lipases and/or their (co-)regulators. Starting from *in silico* analysis, we identified and substantiated miR-124a, a miRNA previously reported to promote microglia quiescence and early neurogenesis [16,17,18], as a regulator of TG catabolism via ATGL and CGI-58.

## 2. Results

### 2.1. miR-124a Regulates Expression of Adipose Triglyceride Lipase (ATGL) and Comparative Gene Identification 58

Here we describe the first steps toward the characterization of the role of miR-124a in the regulation of lipolysis. We performed *in silico* analysis to identify miRNAs with sequence complementarity to the main lipases and their co-regulators thus allowing regulation of lipolysis. We screened the complete set of miRNAs (miRBase 10.0 database), but, interestingly, only one miRNA listed in the database, miR-124a, showed a significant sequence match to the 3' untranslated regions (UTRs) of the murine *Atgl* and *Cgi-58* (mRNAs) (Figure S1A). The 3'UTRs of murine *Hsl*, *Mgl* and *G0s2* (mRNAs) did not contain potential target sequences for miR-124a. Using RNA hybrid [19], the theoretical free energy of the interaction of miR-124a with the *Atgl* and *Cgi-58* target sequences were calculated to be −27.6 and −20.8 kcal/mol, respectively (Figure S1B,C). Global 3'UTR sequence alignment by clustalx [20] showed that the seed sequence of murine *Atgl* and *Cgi-58* (mRNAs) is conserved across many species (Figure S1D,E).

**Figure 1:**
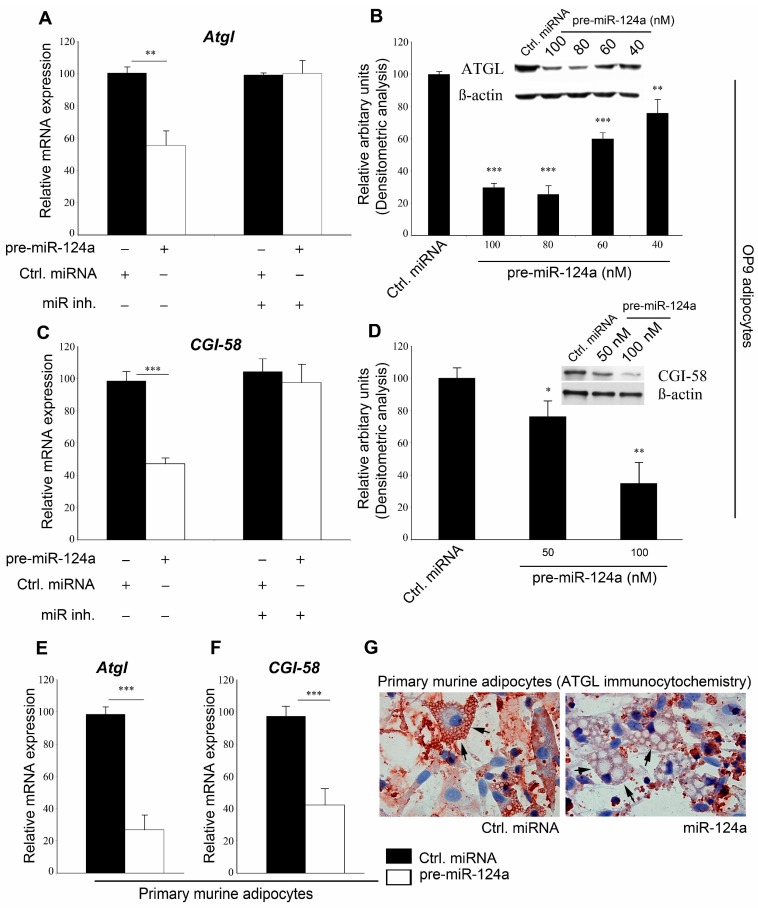
Micro-RNA 124a (miR-124a) regulates expression of adipose triglyceride lipase (ATGL) and *CGI-58*. (**A**) pre-miR-124a or control pre-miRNA was co-transfected with or without miR-124a inhibitor in OP9 adipocytes and mRNA- (**A**) or protein-expression (**B**) of *Atgl* was determined; (**C**,**D**) *Cgi-58* (mRNA)- (**C**) or protein-expression (**D**) was analyzed after co-transfecting pre-miR-124a or control pre-miRNA with or without miR-124a inhibitor into OP9 adipocytes; (**E**–**G**) Lentiviral particles carrying either control pre-miRNA or miR-124a were transduced into primary mouse adipocytes and qRT-PCR was performed for *Atgl* (mRNA) (**E**) or *Cgi-58* (mRNA) (**F**); Immunocytochemical analysis highlights the lipid bound “active pool” of ATGL. Arrows depict ATGL staining on lipid droplets; (**G**) Data are shown as average ± standard deviation and represent three independent experiments. *** *p* < 0.001, ** *p* < 0.01, * *p* < 0.05.

To test for biological relevance of these findings, we analyzed alterations in response to miR-124 on the levels of *Atgl* and *Cgi-58* (mRNA) and protein expressions as well as their lipolytic activities. Because *Atgl* expressed strongest in adipocytes, we chose the easily adipogenic-differentiated OP9 model cell line for our experiments [21]. OP9 adipocytes were transfected with either pre-miR-124a or control pre-miRNA. Seventy-two hours after transfection, adipocytes were harvested and analyses were performed. Quantitative real time polymerase chain reaction (qRT-PCR) using *Atgl* (mRNA) specific primers revealed that miR-124a down-regulates *Atgl* (mRNA), on average, by 44.6%. This reduction of *Atgl* (mRNA) levels in miR-124a treated cells could be reversed using miR-124a inhibitor (Figure 1A) demonstrating that the effect on *Atgl* (mRNA) was specific for miR-124a.

Western blot (WB) analyses revealed that miR-124a transfection resulted in a dose dependent down-regulation of cellular ATGL protein levels (Figure 1B) that was even more pronounced than its effect on *Atgl* (mRNA). Again, the response could be abrogated by the specific miR-124a inhibitor (Figure S2A,B). In addition to ATGL, miR-124a also suppressed CGI-58 expression. Transfection of OP9 adipocytes with pre-miR-124a resulted in a decrease of the cellular *Cgi-58* (mRNA) level by 52% compared to cells transfected with control pre-miRNA (Figure 1C). Protein expression of CGI-58 was, on average, reduced by 24% and 65% when OP9 adipocytes were treated with 50 and 100 nM miR-124a, respectively (Figure 1D), which was blocked by miR-124 inhibitor, once more, arguing for the specificity of the process (Figure 1C, Figure S2C,D).

To confirm these observations in a more physiological model, mouse primary adipocytes were transduced with lentiviral particles expressing either pre-miR-124a or control pre-miRNA. As shown in Figure 1E,F, miR-124a transduction resulted, on average, in a 73% and 56% decrease of *Atgl* and *Cgi-58* (mRNA) levels, respectively, compared to control (Figure 1E,F). Immunohistochemical analysis showed a significant reduction of ATGL on lipid droplets in pre-miR124a treated cells compared to cells treated with control pre-miRNA (Figure 1G). Transfection of pre-miR-124a, however, did not change the mRNA expression of *Hsl*, *G0s2*, *Mgl* or *Perilipin A* (mRNAs) (Figure S2E).

### 2.2. miR-124a Regulates ATGL and CGI-58 via Single Targeting Sequences in Their 3'UTRs

**Figure 2:**
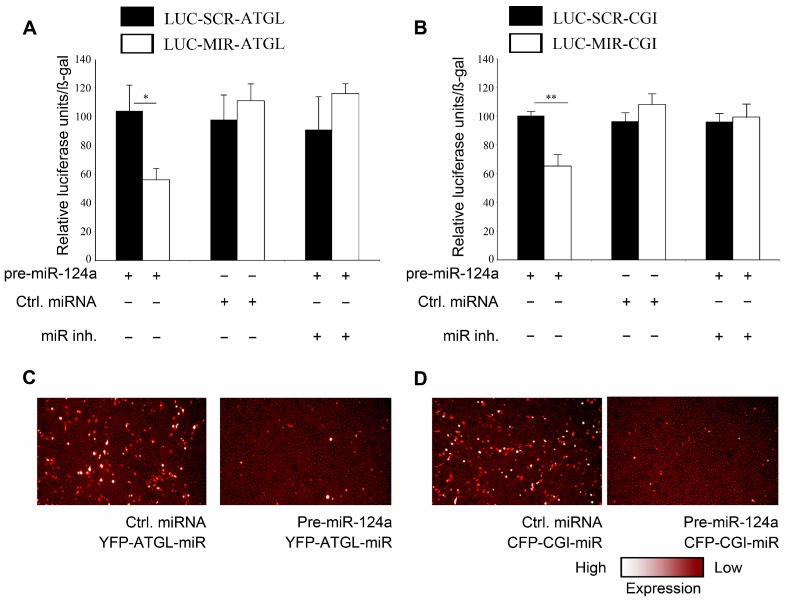
miR-124a interacts with the 3'UTR of *Atgl* (mRNA) and *Cgi-58* (mRNA). (**A**) Luciferase activity (relative units, normalized to β-gal activity for transfection normalization) measured after co-transfection of HeLa cells with either LUC-MIR-ATGL or LUC-SCR-ATGL with control pre-miRNA or pre-miR-124a and miR-124a inhibitor as indicated; (**B**) LUC-MIR-CGI or LUC-SCR-CGI was co-transfected into HeLa cells together with either miR-124a or control pre-miRNA and miR-124a inhibitor. β-gal containing plasmid was transfected for transfection normalization. Values are presented as relative luciferase units normalized to β-gal activity; (**C**,**D**) HeLa cells were co-transfected with YFP-ATGL-miR (**C**) or CFP-CGI-miR (**D**) and either pre-miR-124a or control pre-miRNA. Data are shown as average ± standard deviation and represent three independent experiments. ** *p* < 0.01, * *p* < 0.05.

Subsequently, we tested if miR-124a regulates *Atgl* and/or *Cgi58* (mRNAs) via the predicted target sequences. Therefore, we first introduced the predicted 23 bp sequence from the 3'UTR of the *Atgl* (mRNA) or a scrambled control sequence into the 3'UTR of a luciferase reporter gene, thus generating LUC-MIR-ATGL and LUC-SCR-ATGL, respectively (Figure S3A). Co-transfection of LUC-MIR-ATGL with pre-miR-124a in HeLa cells resulted in 46% decrease in luciferase activity as compared to co-transfection using control pre-miRNA (Figure 2A). In line with our previous results, miR-124a inhibitor abolished this effect, whereas, co-transfection of LUC-SCR-ATGL with either pre-miR-124a or ctrl pre-miRNA did not lead to any significant changes in luciferase activity. This indicates that the miR-124a effect was specific, and dependent on the presence of the suspected target sequence. Furthermore, the presence of the suspected target sequence was sufficient to confer miR-124a dependent suppression of an independent reporter protein. The same approach was used to investigate if miR-124a regulates *Cgi58* (mRNA) via the predicted target sequence (Figure S3B). Upon co-transfection of LUC-MIR-CGI58 with pre-miR-124a luciferase activity was decreased by 34.8% compared to control pre-miRNA (Figure 2B). Again, the miR-124a inhibitor abolished the reduction of luciferase activity and activity of LUC-SCR-CGI58 was not affected under any of the conditions tested. This, again, indicated that the effect of miR-124a was specific, dependent on the suspected target sequence, and sufficient to suppress an independent reporter protein.

To independently assess the direct effect of miR-124a on its respective target sequences, we used a heterologous hybrid system. We cloned the potential regulatory region of the *Atgl* 3'UTR into a CMV promoter controlled ATGL-YFP (Yellow Fluorescence Protein) expression plasmid (YFP-ATGL-miR, Figure S3C). Co-transfection of YFP-ATGL-miR with pre-miR-124a, showed a pronounced down-regulation of YFP fluorescence compared to co-transfection with control pre-miRNA (Figure 2C). Similarly, co-transfection of pre-miR-124a with a CMV promoter controlled CGI58-CFP containing the putative miR-124a interaction site in its 3'UTR (CFP-CGI-miR, Figure S3D) reduced CFP fluorescence compared to co-transfection with control pre-miRNA (Figure 2D).

*In silico* analysis revealed a single miR-124a target site in the 3'UTRs of the *Atgl* and *Cgi-58* (mRNAs). In order to investigate if miR-124a regulates either of them via any other site(s) in their respective 3'UTR, we cloned either the original or a “putative target site” mutated 3'UTR of *Atgl* or *Cgi-58* into luciferase reporter plasmids (Figure S3E,F). Subsequently, each of them was either co-transfected with control pre-miRNA or with pre-miR-124a. Luciferase activity of a reporter plasmid containing the *wt* 3'UTR of *Atgl* was reduced by ~60% upon pre-miR-124a co-transfection, as compared to control pre-miRNA co-transfection (Figure S3G) in HeLa cells. Similarly, luciferase activity of a reporter plasmid containing the *wt* 3'UTR of *Cgi-58* was reduced by ~43% when co-transfected with pre-miR-124a, as compared to control-pre-miRNA co-transfection (Figure S3H). Reporter plasmids, carrying the *Atgl* or *Cgi-58* 3'UTRs, mutated at their respective target sites, showed no significant differences, when co-transfected with control-pre-miRNA or pre-miR-124a, in respect to the cellular luciferase activity (Figure S3G,H). Taken together, our results strongly suggest that miR-124a regulates *Atgl* and *Cgi-58* expression via the predicted, respective, single target sites in the 3'UTRs of their mRNAs.

**Figure 3:**
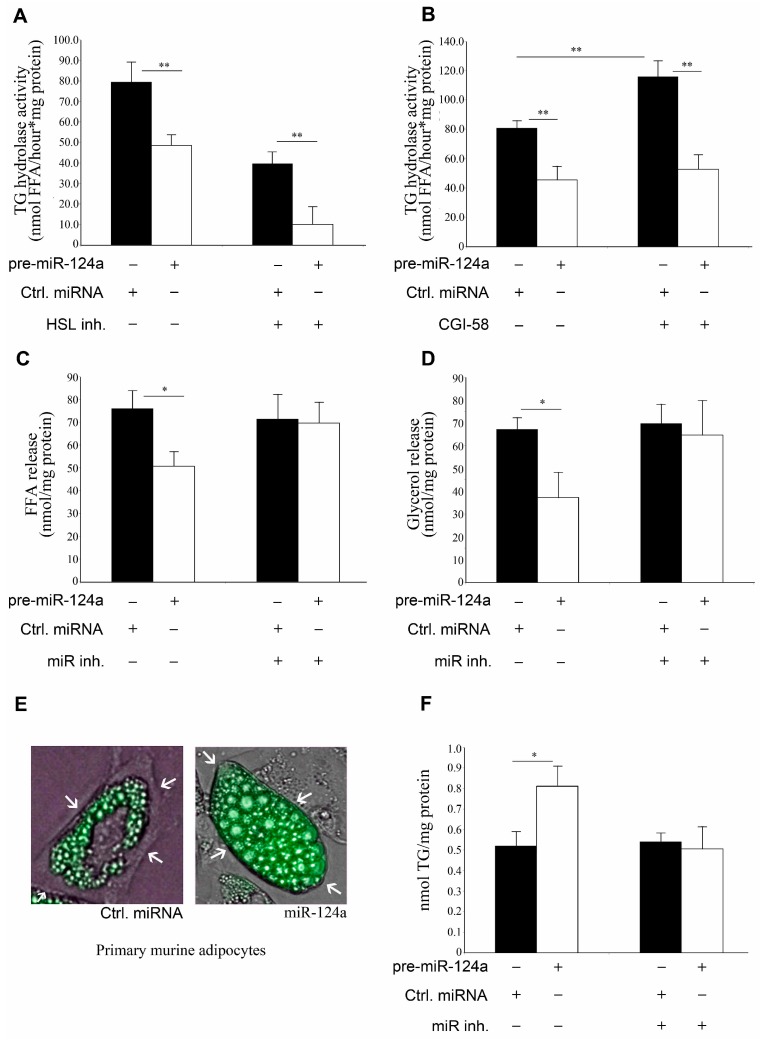
miR-124a negatively regulates lipolysis (**A**,**B**) OP9 adipocytes were transfected Z787632 with either control pre-miRNA or pre-miR-124a. TG hydrolase activities were analyzed in the presence or in the absence of 76-0079 (HSL inhibitor) (**A**) or 500 ng recombinant CGI-58 protein (**B**); (**C**,**D**) free fatty acid (**C**) and glycerol (**D**) release was quantified in OP9 adipocytes after transfection with either pre-miR-124a or control pre-miRNA with or without miR-124a inhibitor; (**E**) Primary mouse adipocytes transduced with lentiviral particles carrying either control pre-miRNA or miR-124a were stained with BODIPY 493/503 fluorescence dye; White arrows point towards the differences in lipid droplet size (**F**) OP9 adipocytes were transfected with either pre-miR-124a or control pre-miRNA with or without miR-124a inhibitor and cellular triglyceride (TG) was quantified. Data are shown as average ± standard deviation and represent three independent experiments. ** *p* < 0.01, * *p* < 0.05.

### 2.3. miR-124a Down-Regulates Lipolysis

To determine a possible functional role of miR-124a in lipolysis we monitored cellular TG hydrolase activity in pre-miR-124a and control pre-miRNA transfected OP9 adipocytes. To distinguish between ATGL and HSL activities, we performed lipase activity assays in the presence or absence of the specific HSL inhibitor, 76-0079 [14,22]. On average, we observed a 39% reduction in total lipase activity and a 74% reduction in non-HSL lipase activity, which were attributable to pre-miR-124a transfection (Figure 3A). Since it was found that in adipocytes >90% of the TG hydrolase activity is mediated by ATGL and HSL [3,22], non-HSL activity essentially equals ATGL activity. Therefore, decreased lipolysis in miR-124a transfected OP9 adipocytes can be mainly attributed to reduction of ATGL and its activity. TG hydrolysis assays in presence or absence of recombinant CGI-58 showed that miR-124a decreased the lipase pool, which is able to respond to CGI-58 (Figure 3B). Reduction in TG hydrolase activity was abrogated by miR-124a inhibitor demonstrating a miR-124a specific effect (Figure S4A,B). Concurrently, FA and glycerol release was decreased by ~33% and ~44%, respectively, with pre-miR-124a compared to control pre-miRNA. Decreased lipolysis was abrogated by miR-124a inhibitor (Figure 3C,D). In addition to basal lipolysis, isoproterenol activated lipolysis was also reduced by miR-124a by ~41%, as measured by FA release (Figure S4C). Isoproterenol, however, did not significantly affect the expression of miR-124a in OP9 adipocytes (Figure S4D). Decreased lipolysis in response to miR-124a increased the cellular neutral lipid content compared to adipocytes treated with control pre-miRNA (Figure 3E,F and Figure S4E). Elevated TG concentration could be rescued by transfecting the OP9 cells with *Atgl* plasmid without 3'UTR (Figure S4F). When adipocytes were co-transfected with a miR-124a inhibitor, the TG concentrations were not affected by miR-124a.

### 2.4. Negative Correlation of miR-124a and Atgl Expression in Various Tissues

**Figure 4:**
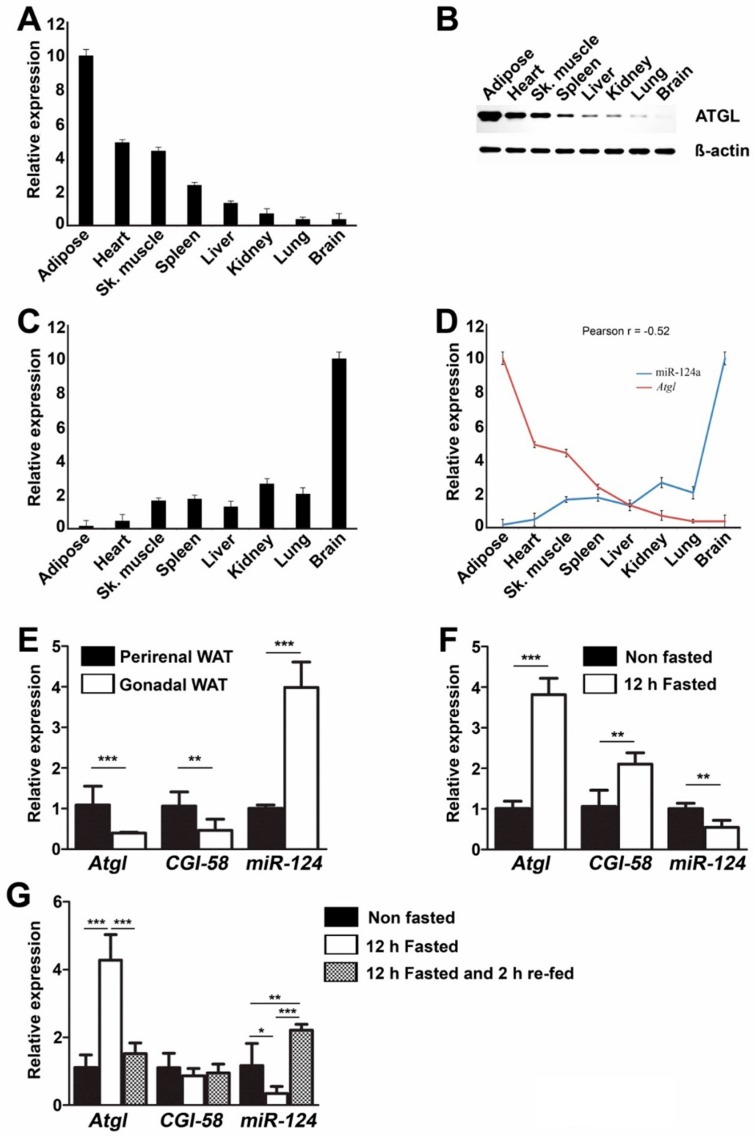
miR-124a expression negatively correlates to *Atgl* (mRNA) levels in various murine tissues. (**A**,**B**) Relative *Atgl* (mRNA) (**A**) and protein (**B**) expression measured in various murine tissues. *Atgl* (mRNA) level in adipose tissue arbitrarily set to 10; (**C**) Relative expression of mature miR-124a measured by qRT-PCR. Mature miR-124a level in brain arbitrarily set to 10; (**D**) Correlation between *Atgl* (mRNA) and miR-124a expression in various murine tissues; (**E**) Relative *Atgl*, *Cgi-58* and miR-124a expression measured in murine perirenal as well as gonadal WAT; (**F**) Relative *Atgl*, *Cgi-58* and miR-124a expression measured in murine perirenal WAT from *ad libitum* fed or, 12 h overnight fasted, mice; (**G**) Relative *Atgl*, *Cgi-58* and miR-124a expression measured in murine liver from *ad libitum* fed or, 12 h overnight fasted or, 12 h overnight fasted and 2 h re-fed mice. Data are shown as average ± standard deviation and represent three independent experiments (**A**–**D**) or five independent experiments (**E**–**G**). *** *p* < 0.001, ** *p* < 0.01, * *p* < 0.05.

We analyzed miR-124a and *Atgl* (mRNA) as well as ATGL protein expression in various murine tissues using qPCR and WB, respectively. Interestingly, we found a strong negative correlation (Pearson *r* = −0.52) between miR124a expression level and *Atgl* (mRNA) abundance. ATGL protein levels across the various tissues corresponded well to the respective *Atgl* (mRNA) levels (Figure 4A–D).

In contrast however, we failed to obtain such a correlation between *Cgi-58* (mRNA) and miR-124a expression levels. This might reflect that *Cgi-58* expression is regulated by various other elements that mask the effect of miR-124a in some of the tissues under physiological condition(s). Interestingly, *Atgl* and *Cgi-58* expression is low in gonadal adipose tissue compared to perirenal adipose tissue (Figure 4E). In contrast to *Atgl* and *Cgi-58* (mRNAs) expressions, miR-124a expression is about four-fold higher in gonadal adipose tissue compared to perirenal adipose tissue (Figure 4E). However, a causal relationship between miR124a levels and *Atgl* (mRNA) and protein concentrations in tissues remains to be investigated.

### 2.5. miR-124a Regulates the Expression of Atgl and Cgi-58 during Fasting Conditions

Both, *Atgl* and *Cgi-58* (mRNAs) expressions were significantly increased in white adipose tissue after 12 h fasting in mice, whereas miR-124a expression was decreased by 50% (Figure 4F). Similarly, *Atgl* (mRNA) expression in liver increased 4-fold after 12 h fasting and subsequently, returned to basal levels after 2 h of re-feeding. miR-124a expression in liver showed an opposite trend during fasting and re-feeding. Expression of miR-124a decreased by ~71% after 12 h fasting and increased by ~85% after 2 h re-feeding. *Cgi-58* (mRNA) expression in murine liver, however, seemed to be unaffected by fasting and re-feeding (Figure 4G). This indicates that miR-124 might play a role in the regulating of ATGL during fasting and re-feeding in murine liver and WAT and thus might be involved in the regulation of changes in lipolysis as well as body metabolism during feeding cycles.

## 3. Experimental Section

### 3.1. Animal Care and Tissue Harvesting

Eight- to ten-week-old mice were anesthetized using isoflurane and subsequently sacrificed either in fed condition, after 12 h overnight fasting or after 12 h overnight fasting and 2 h re-feeding by cervical dislocation. The tissues were rapidly excised and frozen in liquid nitrogen.

### 3.2. Cell Lines and Constructs

OP9 preadipocytes were differentiated to adipocytes by insulin-oleate treatment as described earlier [21]. Primary adipocytes were isolated from gonadal white adipose tissue of C57BL/6 mice as described earlier [23].

Putative miR-124a target sequences from *Atgl*, *Cgi-58*’s 3'UTR, or scrambled control were cloned into the 3'UTR of a luciferase gene, which was under the control of a CMV promoter. Additionally the sequences were cloned in the 3'UTR of CGI-58-CFP or ATGL-YFP (kind gift from Martina Schweiger). Luciferase reporter plasmid carrying wt or mutated *Atgl* or *Cgi-58* 3'UTR regions were purchased from Origene (Origene, MD, USA) (Figure S3E,F).

### 3.3. Transfection and Lentiviral Transduction

Transient transfections were performed with the respective plasmids (3 µg/well of a 6-well plate) and pre-miRNAs (100 nM or as stated in Figure 1; PM10691, Ambion, Austin, TX, USA). Additionally, we used single-stranded, modified RNA designed to specifically inhibit selected endogenous miR-124a referred to in this manuscript as miRNA inhibitor (100 nM; AM10691, Ambion) or controls (100 nM; AM17-010) using Lipofectamine 2000 (Invitrogen, Waltham, MA, USA) according to company’s protocol. Lentiviral particles carrying either pre-miR-124 or control pre-miRNA were obtained from GeneCopoeia (Rockville, MD, USA). Primary adipocytes were transduced with the lentiviral particles (10^4^ TU/mL) along with polybrene (8 µg/mL, Sigma) and analyzed after 72 h.

### 3.4. Triacylgycerol Hydrolase Assay

TG hydrolase assay was performed as described earlier (Lass *et al.*, 2006). In short, adipocytes were homogenized in lysis buffer and lipid-free cytosolic fraction was used for TG hydrolase assays. Non-labeled triolein (Sigma Aldrich, St. Louis, MO, USA) and [9,10-3H(N)-triolein] (American Radiolabeled Chemicals (ARC), St. Louis, MO, USA) emulsified with phosphatidylcholine/phosphatidylinositol (Sigma Aldrich) was used as the substrate. HSL inhibitor 76-0079 (NNC 0076-0000-0079, Novo Nordisk, Denmark) was used to assess ATGL activity.

### 3.5. Free Fatty Acid and Glycerol Release Assay

Adipocyte conditioned medium was analyzed for FFA and glycerol content (release) by commercial kits (Wako Chemicals, Neuss, Germany and Sigma-Aldrich, respectively).

### 3.6. Intracellular TG Measurement

Intracellular lipids were extracted with hexan:isopropanol (3:2; *v*/*v*), dried under N_2_ gas and solubilized in 1% triton X-100 with sonication. TG concentration was determined using TG FS kit (Diasys, Holzheim, Germany).

### 3.7. Microscopic Analysis of Adipocytes

Adipocytes were stained using Bodipy 493/503 (Invitrogen) and analyzed by fluorescence microscopy with green filter.

### 3.8. Flow Cytometry

The amount of intracellular bodipy staining (Invitrogen) was analyzed by flow cytometry performed on a FACSAria flow cytometer using FACSDiva 6 software for data acquisition (BD Biosciences, Franklin Lakes, NJ, USA). The median intensity values were plotted for statistical analysis using FCS Express software (*De Novo*).

### 3.9. Luciferase Assay

Firefly luciferase activity was measured by One-glo system (Promega, Fitchburg, WI, USA) and was normalized to β-gal activity measured by β-glo (Promega) according to company’s protocol.

### 3.10. Western Blot and Immunohistochemistry

Protein was extracted using RIPA lysis buffer (Sigma) supplemented with protease inhibitor cocktail (Roche, Basel, Switzerland). Antibodies used: ATGL (2138, Cell Signaling, Danvers, MA, USA), CGI-58 (H00051099-M01, Abnova, Taipei City, Taiwan), β-actin (sc-47778, Santa Cruz, CA, USA). The resultant bands were analyzed densitometrically using ImageJ. ATGL antibody (10006409, Cayman, MI, USA) was used for immunocytochemistry and detected using DAKO AEC detection system (DAKO, Glostrup, Denmark).

### 3.11. qRT-PCR

Total RNA was isolated using Trizol reagent (Invitrogen) and cDNA was prepared using high capacity reverse transcription kit (Applied Biosystems, Waltham, MA, USA). qRT-PCRs were performed using Taqman Universal PCR master mix (Applied Biosystems). Primer sequences are provided in Table S1. miR-124a expression was analyzed by primers specific to miR-124 (LNA PCR primer set, Exiqon; Vedbaek, Denmark).

### 3.12. Statistical Methods

Results are presented as mean ± standard deviation (S.D.). Comparisons were performed using unpaired *t*-test and presented as *** for *p* ≤ 0.001, ** for *p* ≤ 0.01, * for *p* ≤ 0.05.

## 4. Discussion

TG lipolysis is mediated by a highly regulated multi-enzyme complex that drives the catabolism of cellular TG stores. Three lipases, ATGL, HSL and MGL, are needed for sequential hydrolysis of TG into glycerol and three FA. ATGL is the enzyme that catalyzes the first, and rate-limiting, step in lipolysis and its activity is regulated by its activator, CGI58, and its inhibitor, G0S2. Additionally, hormonal signaling pathways and lipid droplet-associated factors regulate TG lipolysis [1,24]. During the current study, we identified a putative novel mechanism for the regulation of lipolysis. Our data provide evidence that miR-124a can control the expression of the rate-limiting enzyme in TG lipolysis, ATGL and its co-activator, CGI58. While there seems to exist a correlation between miR-145 [25] expression and *Cgi-58* down-regulation, no such miRNA-based regulation of *Atgl* is currently known, to the best of our knowledge. *Atgl* expression is well regulated during fasting and feeding, at least partially, by transcriptional regulation via insulin signaling [26]. Our results provide evidence of a novel pathway regulating *Atgl* via miR-124a in WAT as well as liver during fasting and re-feeding. This is of special importance considering the requirement of tight regulation of lipid metabolism during fasting and feeding. Since modulation of lipid catabolism is also implicated in various pathological situations, such as insulin resistance or cancer associated cachexia, miR-124a may be considered as a potential drug target for further metabolic research [14].

## Supplementary Materials

Supplementary materials can be found at http://www.mdpi.com/1422-0067/16/04/8555/s1.

## References

[1] Lass A., Zimmermann R., Oberer M., Zechner R. (2011). Lipolysis—A highly regulated multi-enzyme complex mediates the catabolism of cellular fat stores. Prog. Lipid Res..

[2] Ahmadian M., Wang Y., Sul H.S. (2010). Lipolysis in adipocytes. Int. J. Biochem. Cell. Biol..

[3] Lass A., Zimmermann R., Haemmerle G., Riederer M., Schoiswohl G., Schweiger M., Kienesberger P., Strauss J.G., Gorkiewicz G., Zechner R. (2006). Adipose triglyceride lipase-mediated lipolysis of cellular fat stores is activated by CGI-58 and defective in chanarin-dorfman syndrome. Cell. Metab..

[4] Yamaguchi T., Omatsu N., Morimoto E., Nakashima H., Ueno K., Tanaka T., Satouchi K., Hirose F., Osumi T. (2007). CGI-58 facilitates lipolysis on lipid droplets but is not involved in the vesiculation of lipid droplets caused by hormonal stimulation. J. Lipid Res..

[5] Fischer J., Lefevre C., Morava E., Mussini J.M., Laforet P., Negre-Salvayre A., Lathrop M., Salvayre R. (2007). The gene encoding adipose triglyceride lipase (PNPLA2) is mutated in neutral lipid storage disease with myopathy. Nat. Genet..

[6] Haemmerle G., Lass A., Zimmermann R., Gorkiewicz G., Meyer C., Rozman J., Heldmaier G., Maier R., Theussl C., Eder S. (2006). Defective lipolysis and altered energy metabolism in mice lacking adipose triglyceride lipase. Science.

[7] Radner F.P., Streith I.E., Schoiswohl G., Schweiger M., Kumari M., Eichmann T.O., Rechberger G., Koefeler H.C., Eder S., Schauer S. (2010). Growth retardation, impaired triacylglycerol catabolism, hepatic steatosis, and lethal skin barrier defect in mice lacking comparative gene identification-58 (CGI-58). J. Biol. Chem..

[8] Schweiger M., Lass A., Zimmermann R., Eichmann T.O., Zechner R. (2009). Neutral lipid storage disease: Genetic disorders caused by mutations in adipose triglyceride lipase/PNPLA2 or CGI-58/abhd5. Am. J. Physiol.-Endocrinol. Metab..

[9] Albert J.S., Yerges-Armstrong L.M., Horenstein R.B., Pollin T.I., Sreenivasan U.T., Chai S., Blaner W.S., Snitker S., O’Connell J.R., Gong D.W. (2014). Null mutation in hormone-sensitive lipase gene and risk of type 2 diabetes. N. Eng. J. Med..

[10] Taschler U., Radner F.P., Heier C., Schreiber R., Schweiger M., Schoiswohl G., Preiss-Landl K., Jaeger D., Reiter B., Koefeler H.C. (2011). Monoglyceride lipase deficiency in mice impairs lipolysis and attenuates diet-induced insulin resistance. J. Biol. Chem..

[11] Haemmerle G., Zimmermann R., Strauss J.G., Kratky D., Riederer M., Knipping G., Zechner R. (2002). Hormone-sensitive lipase deficiency in mice changes the plasma lipid profile by affecting the tissue-specific expression pattern of lipoprotein lipase in adipose tissue and muscle. J. Biol. Chem..

[12] Osuga J., Ishibashi S., Oka T., Yagyu H., Tozawa R., Fujimoto A., Shionoiri F., Yahagi N., Kraemer F.B., Tsutsumi O. (2000). Targeted disruption of hormone-sensitive lipase results in male sterility and adipocyte hypertrophy, but not in obesity. Proc. Natl. Acad. Sci. USA.

[13] Buchebner M., Pfeifer T., Rathke N., Chandak P.G., Lass A., Schreiber R., Kratzer A., Zimmermann R., Sattler W., Koefeler H. (2010). Cholesteryl ester hydrolase activity is abolished in HSL macrophages but unchanged in macrophages lacking KIAA1363. J. Lipid Res..

[14] Das S.K., Eder S., Schauer S., Diwoky C., Temmel H., Guertl B., Gorkiewicz G., Tamilarasan K.P., Kumari P., Trauner M. (2011). Adipose triglyceride lipase contributes to cancer-associated cachexia. Science.

[15] Parra P., Serra F., Palou A. (2010). Expression of adipose micrornas is sensitive to dietary conjugated linoleic acid treatment in mice. PLoS ONE.

[16] Makeyev E.V., Zhang J., Carrasco M.A., Maniatis T. (2007). The MicroRNA miR-124 promotes neuronal differentiation by triggering brain-specific alternative pre-mRNA splicing. Mol. Cell.

[17] Ponomarev E.D., Veremeyko T., Barteneva N., Krichevsky A.M., Weiner H.L. (2011). MicroRNA-124 promotes microglia quiescence and suppresses EAE by deactivating macrophages via the C/EBP-α-PU.1 pathway. Nat. Med..

[18] Rajasethupathy P., Fiumara F., Sheridan R., Betel D., Puthanveettil S.V., Russo J.J., Sander C., Tuschl T., Kandel E. (2009). Characterization of small RNAs in *Aplysia* reveals a role for miR-124 in constraining synaptic plasticity through CREB. Neuron.

[19] Kruger J., Rehmsmeier M. (2006). Rnahybrid: MicroRNA target prediction easy, fast and flexible. Nucleic Acids Res..

[20] Thompson J.D., Gibson T.J., Higgins D.G. (2002). Multiple sequence alignment using ClustalW and ClustalX. Curr. Protoc. Bioinform..

[21] Wolins N.E., Quaynor B.K., Skinner J.R., Tzekov A., Park C., Choi K., Bickel P.E. (2006). OP9 mouse stromal cells rapidly differentiate into adipocytes: Characterization of a useful new model of adipogenesis. J. Lipid Res..

[22] Schweiger M., Schreiber R., Haemmerle G., Lass A., Fledelius C., Jacobsen P., Tornqvist H., Zechner R., Zimmermann R. (2006). Adipose triglyceride lipase and hormone-sensitive lipase are the major enzymes in adipose tissue triacylglycerol catabolism. J. Biol. Chem..

[23] Fernyhough M.E., Vierck J.L., Hausman G.J., Mir P.S., Okine E.K., Dodson M.V. (2004). Primary adipocyte culture: Adipocyte purification methods may lead to a new understanding of adipose tissue growth and development. Cytotechnology.

[24] Nielsen T.S., Jessen N., Jorgensen J.O., Moller N., Lund S. (2014). Dissecting adipose tissue lipolysis: Molecular regulation and implications for metabolic disease. J. Mol. Endocrinol..

[25] Lin Y.Y., Chou C.F., Giovarelli M., Briata P., Gherzi R., Chen C.Y. (2014). KSRP and microRNA 145 are negative regulators of lipolysis in white adipose tissue. Mol. Cell. Biol..

[26] Chakrabarti P., Kandror K.V. (2011). Adipose triglyceride lipase: A new target in the regulation of lipolysis by insulin. Curr. Diabetes Rev..

