# Supplementary material for: Micro RNA-124a Regulates Lipolysis via Adipose Triglyceride Lipase and Comparative Gene Identification 58
Source: Int J Mol Sci. 2015 Apr 16;16(4):8555–68. doi: 10.3390/ijms16048555 (PMC4425096; doi:10.3390/ijms16048555)
Supplement: Supplementary file 1 [file ijms-16-08555-s001.pdf]

Supplementary Information

Table S1. Primers used.

Gene	Forward Primer	Reverse Primer
Atgl	TCGTGTTTCAGACGGAGAGAA	CAGACATTGGCCTGGATGAG
Cgi-58	TTGCAGGACCTTTTGGGTTA	TCACCGTGTCATCTTCAAACA
G0S2	TCTCTTCCCACTGCACCCTA	TCAGCTCCTGCACACTTTCC
Mgl	GGTCAATGCAGACGGACAGT	AGCTCCATGGGACACAAAGA
Perilipin	CCACCTGGAGGAAAAGATCC	CCACCTGGAGGAAAAGATCC

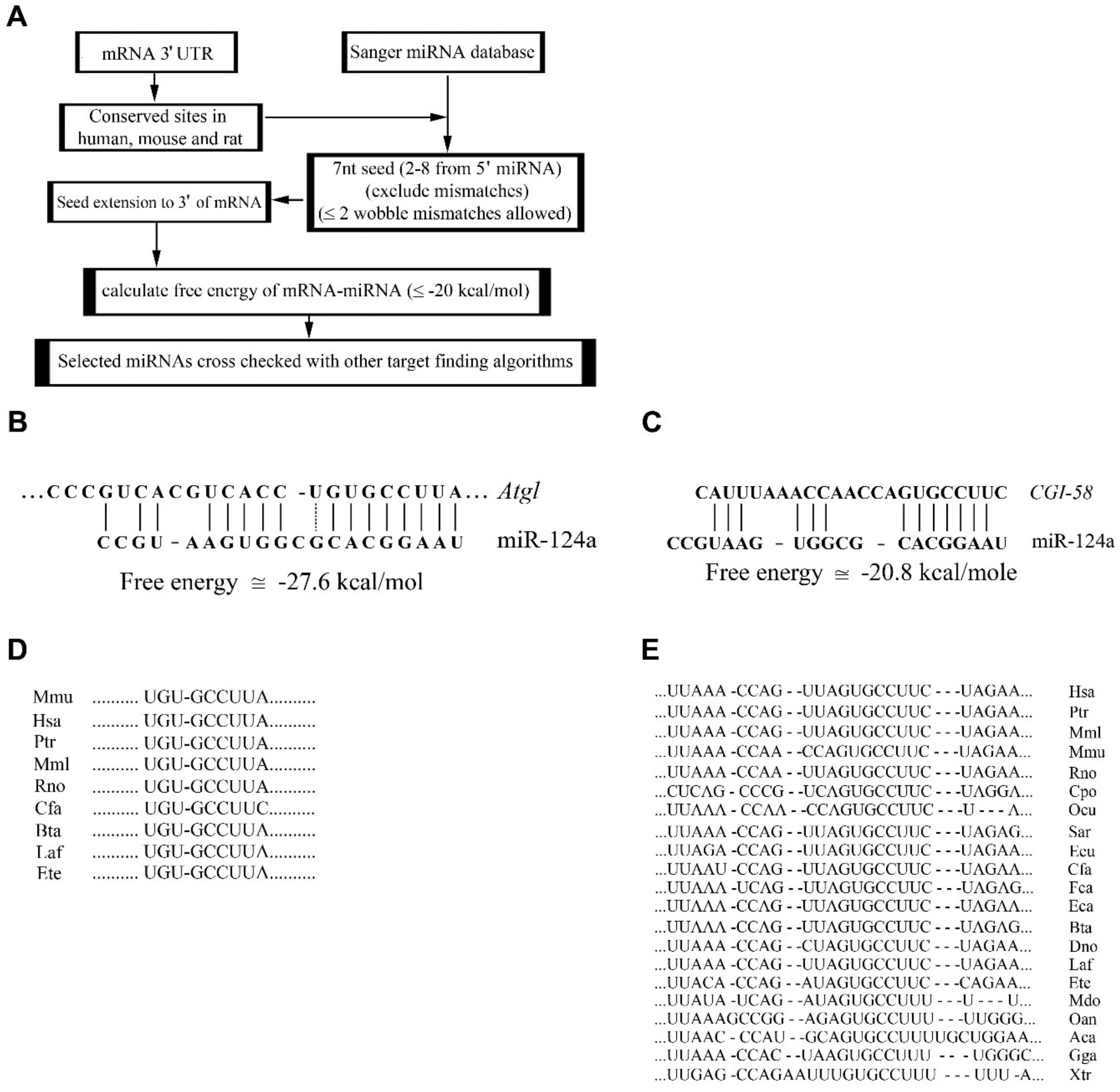

Figure S1. *In silico* analysis for miRNAs targeting ATGL and CGI-58 (also see Figure 1). (**A**) Algorithm used to search for a potential miRNA regulating the expression of lipases and regulatory proteins; (**B**,**C**) miR-124a has a potential target region in the 3'UTR of *Atgl* (**B**) and *Cgi-58* (**C**). Free energy of binding was calculated by RNA-hybrid; (**D**,**E**) The seed sequence in the 3'UTR of *Atgl* (**D**) as well as *Cgi-58* (**E**) is conserved across many species as shown by Clustalx global alignment analysis.

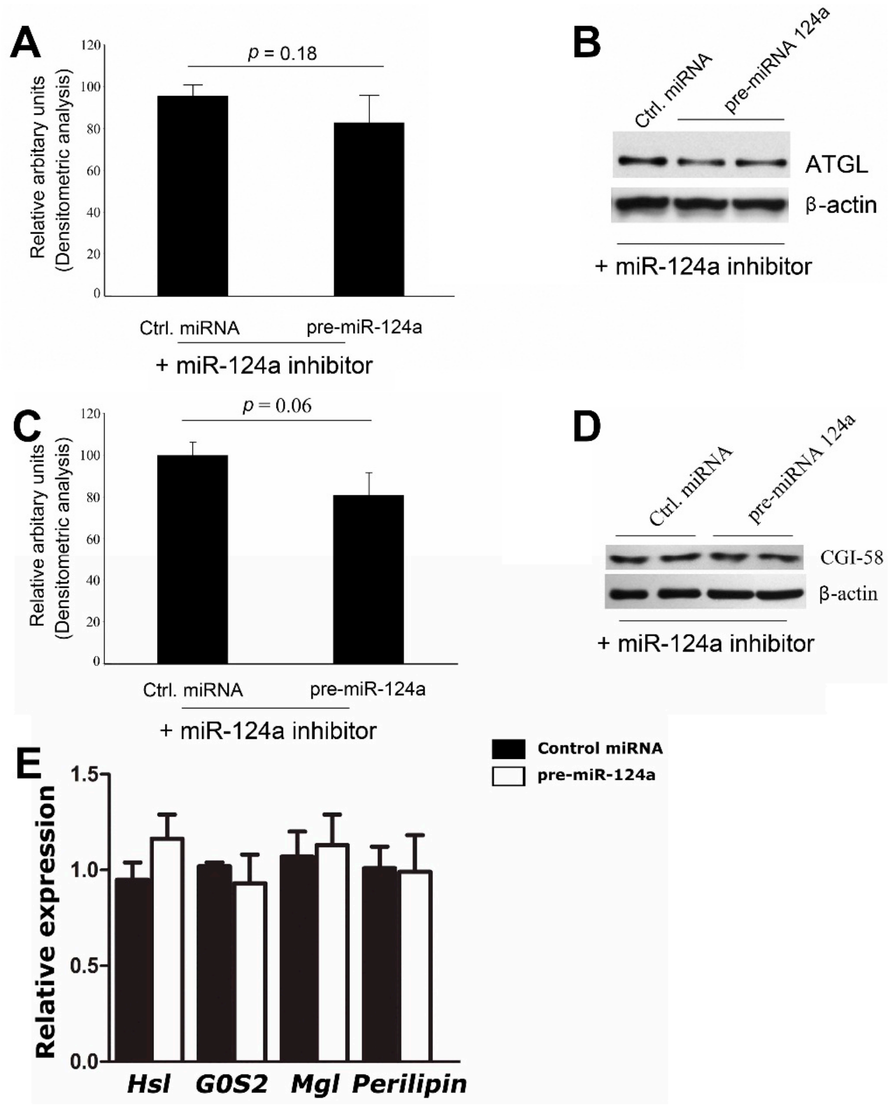

Figure S2. mir-124a specifically alter the expression of ATGL and CGI-58 without affecting the expression of other genes involved in lipid metabolism (Also see Figure 1). (**A**,**B**) ATGL western blot (**B**) analysis was performed from protein lysates of differentiated OP9 adipocytes 60 h after co-transfection with 100 nM pre-miR-124a or control miRNA and miR-124a inhibitor. The graph represents the densitometric analysis (**A**) of bands normalized to β-actin; (**C**,**D**) CGI-58 western blot (**D**) analysis was performed from protein lysates of differentiated OP9 adipocytes 60 h after co-transfection with 100 nM pre-miR-124a or control miRNA and miR-124a inhibitor. The graph represents the densitometric analysis (**C**) of bands normalized to β-actin; (**E**) mRNA expression of *Hsl*, *G0s2*, *Mgl* and *Perilipin* measured after 60 h of transfecting pre-miR-124a or control miRNA in OP9 adipocytes. Data are shown as average ± standard deviation and represent 3 independent experiments.

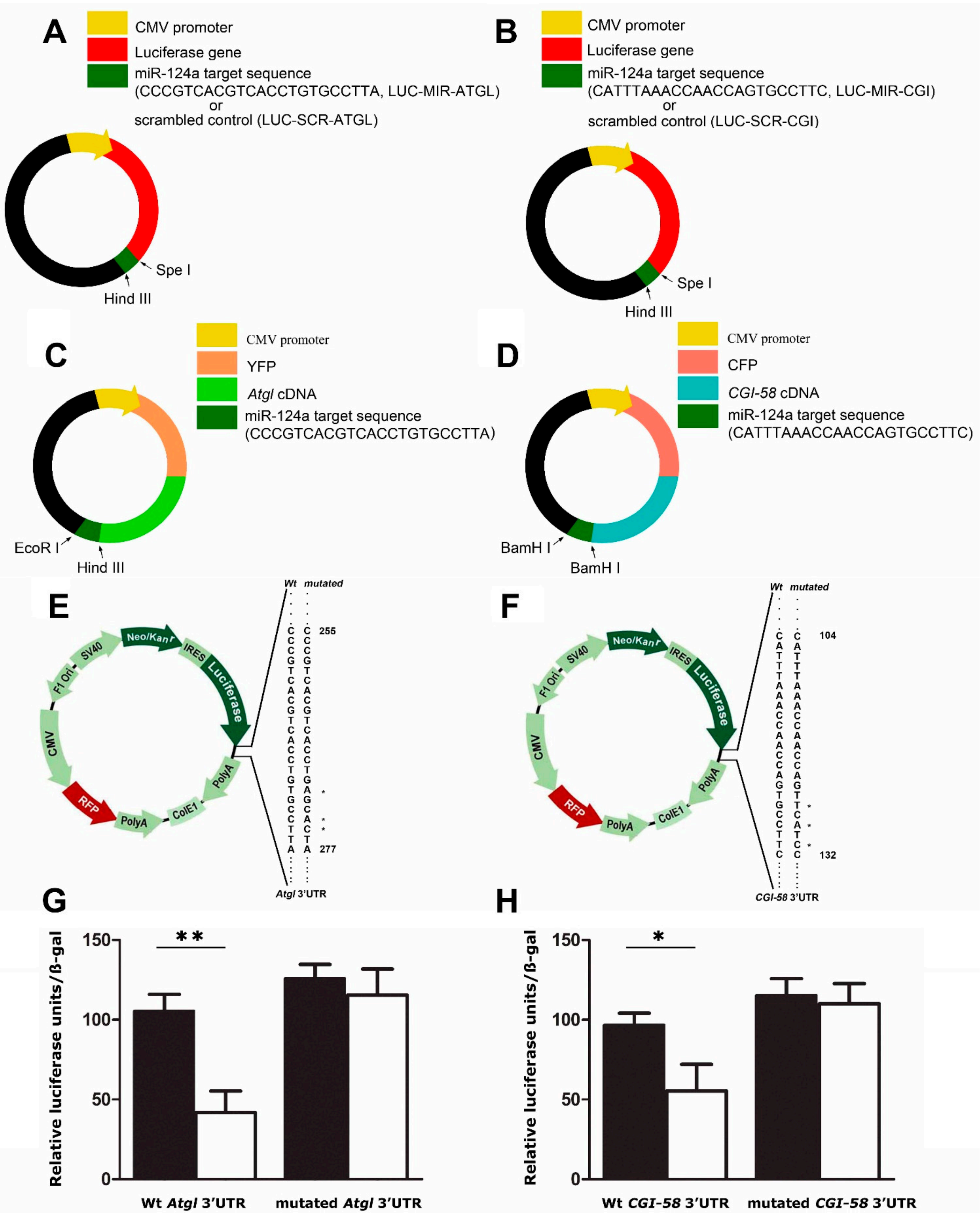

Figure S3. miR-124a regulates ATGL and CGI-58 expression via direct interaction with putative target site present in their respective 3'UTR (Also see Figure 2). (**A**) Vector diagram representing the plasmid construct containing the putative miR-124a target region from the *Atgl* 3'UTR (LUC-MIR-ATGL) or scrambled control (LUC-SCR-ATGL) in the 3'UTR of a luciferase gene; (**B**) Vector diagram representing the plasmid construct containing the

putative miR-124a target region from the *Cgi-58* 3'UTR (LUC-MIR-CGI) or scrambled control (LUC-SCR-CGI) in the 3'UTR of a luciferase gene; (**C**) Vector diagram representing the plasmid construct consisting of the putative miR-124a target region from *Atgl* 3'UTR in the 3’UTR of *Atgl* tagged with YFP (YFP-ATGL-miR); (**D**) Vector diagram representing the plasmid construct consisting of the putative miR-124a target region from 3'UTR of *Cgi-58* in the 3'UTR of *Cgi-58* tagged with CFP (CFP-CGI-miR); (**E**) Vector diagram representing the plasmid construct consisting of wild type or mutated (3 nucleotides mutated in the seed sequence represented by *) 3'UTR *Atgl*; (**F**) Vector diagram representing the plasmid construct consisting of wild type or mutated (3 nucleotides mutated in the seed sequence represented by *) 3'UTR *Cgi-58*; (**G**) Luciferase activity (relative units, normalized to β-gal activity) measured after 60 h of co-transfection of HeLa cells with luciferase reporter plasmid carrying *wt* 3'UTR or mutated (3 nucleotides mutated in the seed sequence) 3'UTR of *Atgl* along with control miRNA or pre-miR-124a; (**H**) Hela cells were co-transfected with luciferase reporter plasmid carrying *wt* 3'UTR or mutated (3 nucleotides mutated in the seed sequence) 3'UTR of *Cgi-58* along with control miRNA or pre-miR-124a and luciferase activity was measured after 60 h. Values are presented as relative luciferase units normalized to β-gal activity. ** $p < 0.01$, * $p < 0.05$.

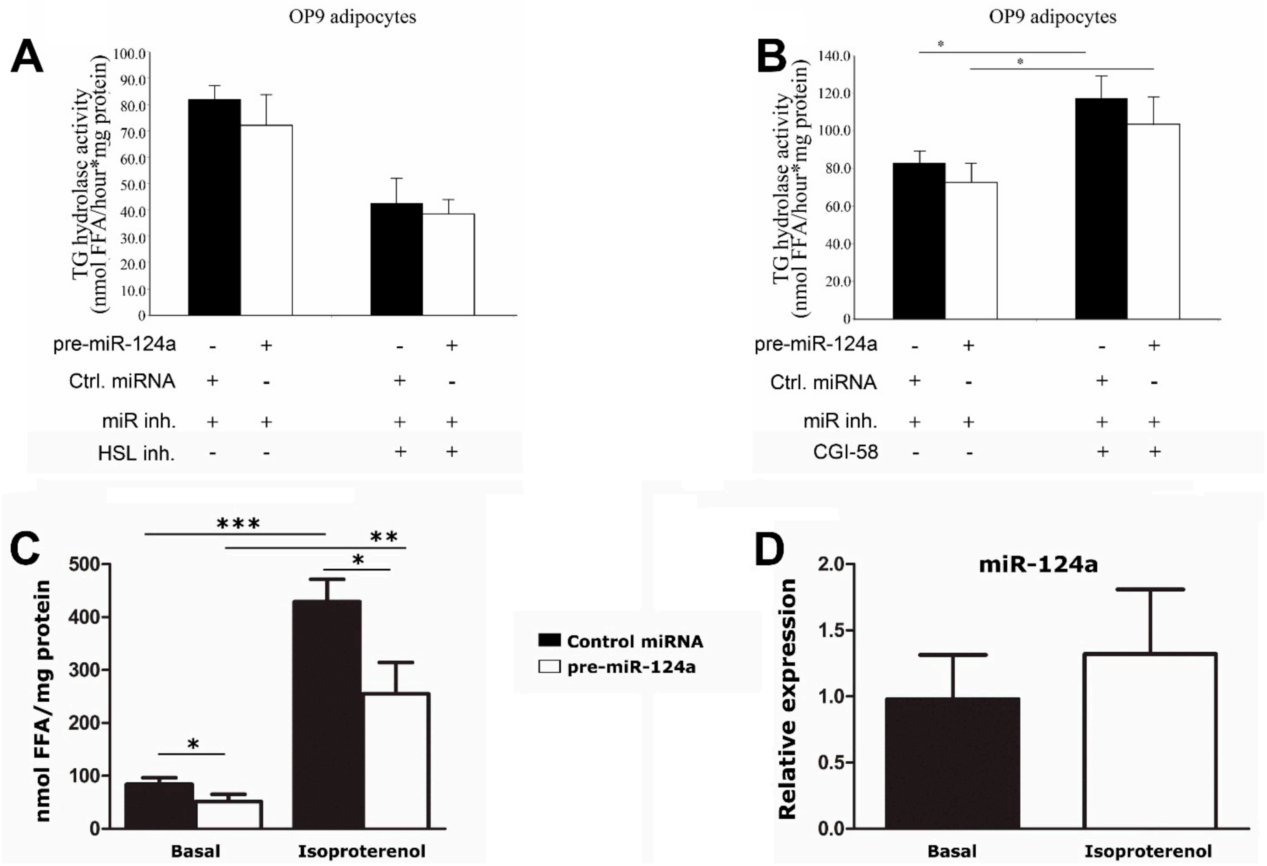

Figure S4. *Cont.*

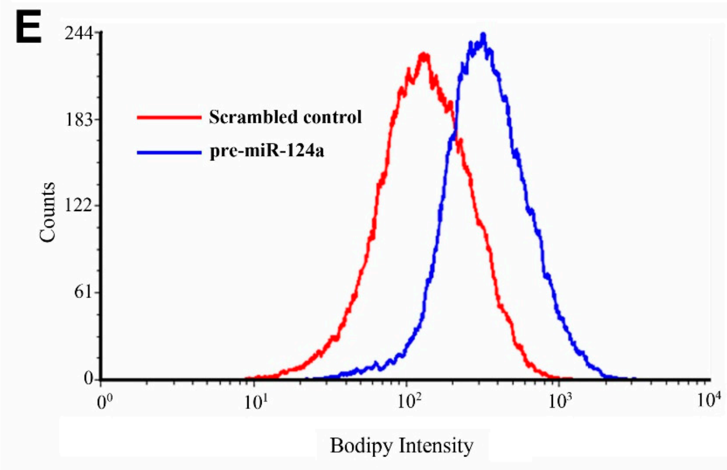

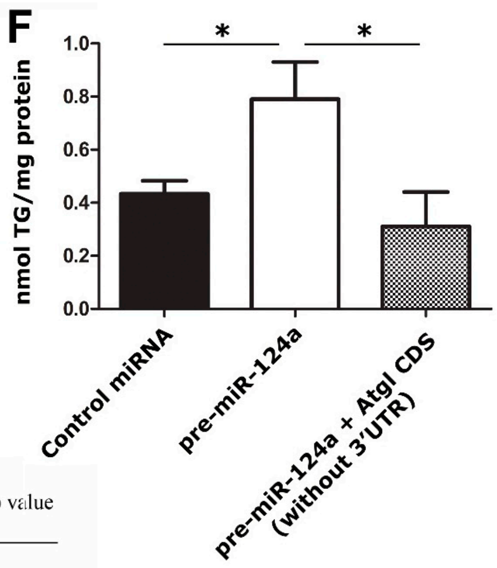

Sample	% of gated cells	Average median bodipy intensity	Standard deviation	p value
OP9 adipocytes + scrambled pre-miRNA (control)	100	153.58	33.99	p = 0.0021
OP9 adipocytes + pre-miR-124a	100	311.90	18.98	

Figure S4. miR-124a decreases TG hydrolysis which can be blocked by using specific inhibitor of miR-124a (Also Figure 3). (**A**) OP9 adipocytes were transfected with either pre-miR-124a or control miRNA with miR-124a inhibitor. TG hydrolase activities from cytosolic protein fractions of adipocytes were analyzed in the presence or in the absence of 76-0079 (HSL inhibitor); (**B**) OP9 adipocytes were transfected with either pre-miR-124a or control miRNA with miR-124a inhibitor. TG hydrolase activities from cytosolic protein fractions of adipocytes were analyzed in the presence or in the absence of recombinant CGI-58 protein; (**C**) OP9 adipocytes were transfected with either pre-miR-124a or control miRNA. Free fatty acid release in the media was quantified by biochemically after 40 h under basal conditions or after 2 h isoproterenol activation and normalized to total protein in cell extracts; (**D**) Relative miR-124a expression in OP9 adipocytes in basal or isoproterenol activated state; (**E**) OP9 adipocytes transfected with either pre-miR-124a or control miRNA and stained with BODIPY 493/503 after 70 h and neutral lipid stores were analyzed by flow cytometry; and (**F**) OP9 adipocytes were transfected with either pre-miR-124a or control miRNA with or without a plasmid carrying *Atgl* coding sequence (without 3'UTR) and cellular triglyceride (TG) was quantified biochemically after 30 h. TG content was normalized to total protein in cell extracts. Data are shown as average ± standard deviation and represent 3 independent experiments. *** $p < 0.001$, ** $p < 0.01$, * $p < 0.05$.

Abbreviations

Aca: Lizard; Bta: Cow; Cfa: Dog; Cpo: Guinea pig; Dno: Armadillo; Eca: Horse; Eeu: Hedgehog; Ete: Tenrec; Fca: Cat; Gga: Chicken; Gga: Chicken; Has: Human; Laf: Elephant; Mdo: Opossum; Mml: Rhesus; Mmu: Mouse; Oan: Platypus; Mml: Rhesus; Mmu: Mouse; Oan: Platypus; Ocu: Rabbit; Ocu: Rabbit; Ptr: Chimpanzee; Rno: Rat; Sar: Shrew; Xtr: Frog.
